# Cardiovascular events in cancer patients with bone metastases—A Danish population‐based cohort study of 23,113 patients

**DOI:** 10.1002/cam4.4027

**Published:** 2021-06-02

**Authors:** Peter H. Asdahl, Jens Sundbøll, Kasper Adelborg, Thomas B. Rasmussen, Anouchka M. Seesaghur, Rohini K. Hernandez, Henrik T. Sørensen, Alma B. Pedersen

**Affiliations:** ^1^ Department of Hematology Aarhus University Hospital Aarhus N Denmark; ^2^ Department of Clinical Epidemiology Aarhus University Hospital Aarhus N Denmark; ^3^ Centre for Observational Research Amgen Ltd Uxbridge UK; ^4^ Centre for Observational Research Amgen Inc Thousand Oaks CA USA

**Keywords:** bone metastasis, cancer, cardiovascular events, cohort, mortality, myocardial infarction, stroke, venous thromboembolism

## Abstract

**Introduction:**

The incidence of cardiovascular events among cancer patients with bone metastases is poorly understood. We examined rates of cardiovascular events among cancer patients with bone metastases and mortality following such events.

**Methods:**

Using Danish health registries, we identified all Danish cancer patients diagnosed with bone metastases (1994–2013) and followed them from bone metastasis diagnosis. We computed incidence rates (IR) per 100 person‐years and cumulative incidence for first‐time inpatient hospitalization or outpatient clinic visit for cardiovascular events, defined as myocardial infarction, ischemic stroke, or venous thromboembolism (VTE). We also analyzed all‐cause mortality rates including cardiovascular events as time‐varying exposure with adjustment for age, sex, and Charlson Comorbidity Index score. All analyses were performed overall and stratified by cancer type (prostate, breast, lung, and other).

**Results:**

We included 23,113 cancer patients with bone metastases. The cumulative incidence of cardiovascular events was 1.3% at 30 days, 3.7% at 1 year, and 5.2% at 5 years of follow‐up. The highest IR was observed for VTE, followed by ischemic stroke and myocardial infarction, both overall and by cancer types. Lung cancer patients with bone metastases had the highest incidence of cardiovascular events followed by prostate and breast cancer. Occurrence of any cardiovascular event was a strong predictor of death (5 years following the event, the adjusted hazard ratio was 1.8 [95% confidence interval: 1.7–1.9]).

**Conclusion:**

Cancer patients with bone metastases had a substantial risk of developing cardiovascular events, and these events were associated with a subsequent increased mortality. Our findings underscore the importance of continuous optimized prevention of and care for cardiovascular disease among cancer patients with bone metastases.

## Graphical abstract

Among 23,113 Danish cancer patients with bone metastases, 4% had developed cardiovascular disease within 1 year from diagnosis. Despite a high risk of cancer related death, cardiovascular disease was associated with a two‐fold increased risk of death.

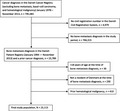

## 1 INTRODUCTION

Bone metastases can occur in most types of cancer, but are most common in breast (4% develop bone metastasis^1^) and prostate cancer (15% develop bone metastasis^2^). At post‐mortem examination up to 70% of these cancer patients have evidence of bone metastasis.^3^ Cancers of the prostate and the breast represent the most common malignant cancers among men and women, respectively.^4^ Lung cancer is also associated with a high risk of bone metastasis.^5^ The development of bone metastasis represents cancer progression, which worsens prognosis and reduces quality of life.^1^, ^2^, ^3^, ^6^ For example, 5‐year survival in breast cancer patients is reduced from 76% to 8% after the occurrence of bone metastasis.^1^

Cancer and cardiovascular disease are the two most common causes of death in developed countries.^7^ The impact of cardiovascular disease on survival in cancer patients is gradually being recognized,^8^ and cardiovascular complications in cancer patients is increasingly common due to long‐term cancer survival.^9^ The risk of cardiovascular disease among cancer patients with bone metastases has been sparsely investigated, but may be increased due to the presence of a number of risk factors, i.e., a state of general inflammation,^10^ reduced physical activity,^11^ more prothrombotic tumor tissue,^12^ and treatment with cardiotoxic agents^13^ and radiotherapy.^14^ The combination of these risk factors may not only increase the risk of cardiovascular disease but could also increase the mortality following cardiovascular disease. On the other hand, several cancer patients receive thromboprophylaxis which may reduce the risk of cardiovascular events.

Using population‐based healthcare registry data from Denmark, we examined the incidence of cardiovascular events, defined as myocardial infarction, ischemic stroke, and venous thromboembolism (VTE), in a cohort of cancer patients with bone metastases. Further, we examined mortality following cardiovascular events.

## 2 METHODS

### 2.1 Design and setting

This population‐based cohort study was based on Danish healthcare data and covers the period from January 1, 1978 to November 30, 2013. The Danish National Health Service secures free and equal access to tax‐supported health care for the entire population.^15^ Complete and valid linkage of all registries at the individual level is possible in Denmark using the unique and ubiquitously used civil registration number assigned to each Danish resident at birth or upon immigration.^16^

### 2.2 Study population

We identified patients with one or more cancer diagnoses (excluding basal cell carcinomas, hematological malignancies, and a sole diagnosis of bone metastasis) registered in the Danish Cancer Registry during 1978–2013. The registry has been operational since 1943 with mandatory reporting of incident cancers to the registry since 1987 and registration according to International Classification of Diseases 10th revision (ICD‐10) coding since 1978. Cancer diagnoses are registered according to the ICD, 7th and 10th revisions. Diagnoses in the Danish Cancer Registry have high accuracy, with 95%–98% completeness and validity of the recorded diagnoses.^17^, ^18^

Patients with a cancer diagnosis were linked to the Danish National Patient Registry covering all Danish hospitals to identify any discharge diagnosis of bone metastasis from January 1, 1994 to November 30, 2013.^19^ Only patients with a cancer diagnosis registered prior to or on the same day as the bone metastasis diagnosis were included. The Danish National Patient Registry contains information on hospital admissions and outpatient clinic visits. Each hospital contact initiates a record including the civil registration number of the patient, dates of admission and discharge, the primary discharge diagnosis, and any secondary diagnoses according to the ICD, 8th and 10th revisions. The validity of a diagnosis of bone metastasis in the Danish National Patient Registry has been investigated for primary cancer sites of the breast and the prostate gland. Using medical chart review as the reference, the overall sensitivity was 44% for prostate cancer metastasis and 32% for breast cancer metastasis. The positive predictive value was 100% for prostate cancer and 86% for breast cancer.^20^

**FIGURE 1:**
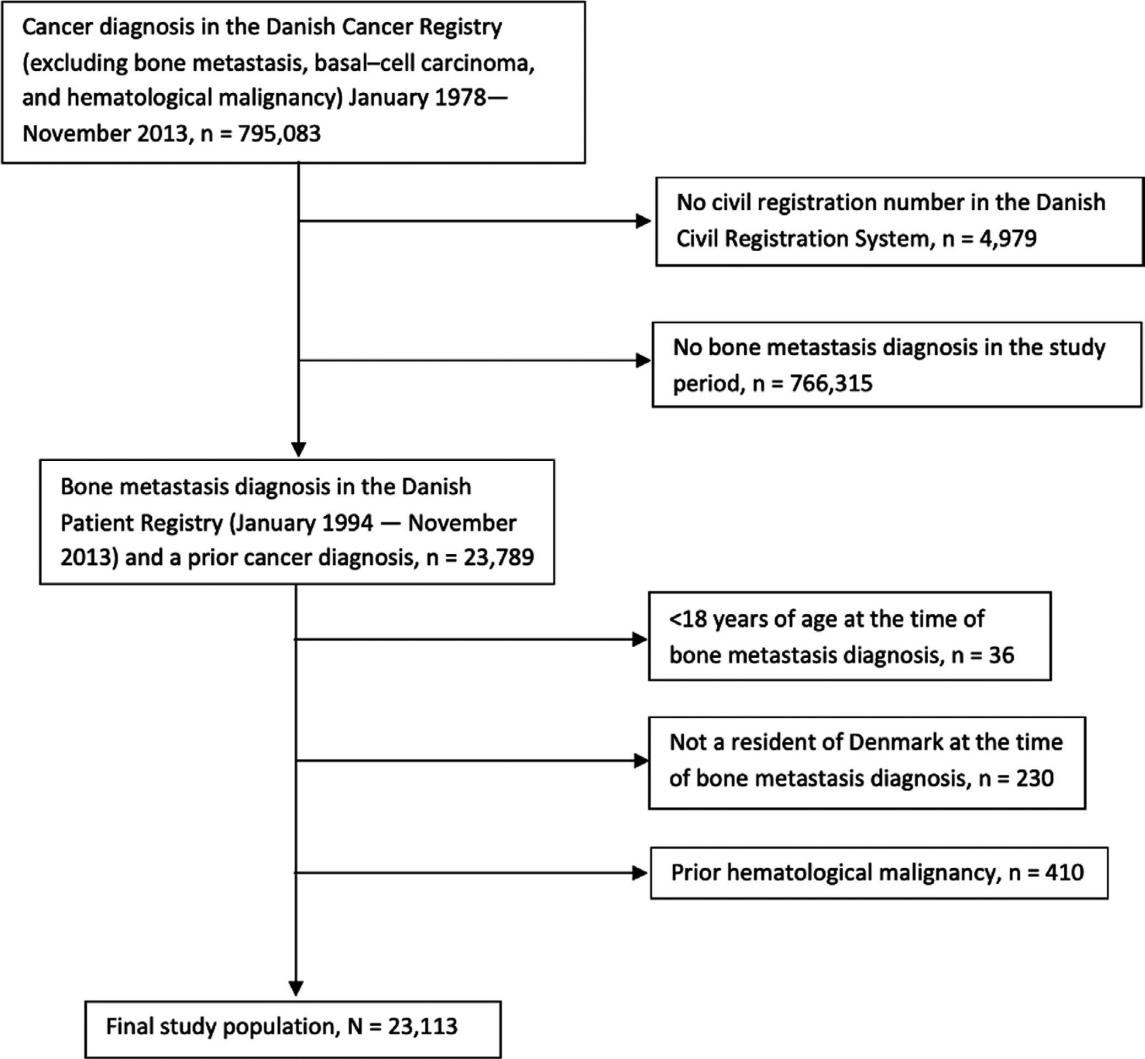
Study flowchart showing the identification of patients from the Danish Cancer Registry and exclusions of patients

We excluded patients aged <18 years, patients residing abroad at the time of bone metastasis diagnosis, and patient with at prior diagnosis of a hematological cancer (Figure 1). The latter exclusion was to make sure the bone metastasis diagnosis was related to the index cancer, and not the hematological cancer. Individuals with cardiovascular disease diagnosed prior to the cancer diagnosis were not excluded. The cohort was analyzed both combined and stratified by cancer diagnosis, including four groups: patients with prostate cancer, breast cancer, lung cancer, and other cancer. In the group that included other cancers, we also included patients with more than one cancer diagnosis.

### 2.3 Cardiovascular events

The cardiovascular events were identified in the Danish National Patient Registry as primary or secondary discharge diagnoses of myocardial infarction, ischemic stroke, or VTE (including deep venous thrombosis or pulmonary embolism). The positive predictive value for myocardial infarction and ischemic stroke in the Danish National Patient Registry is 97%,^21^, ^22^ whereas it is somewhat lower for VTE at approximately 90%.^21^, ^23^ We identified the first‐time inpatient hospitalization or outpatient clinic visit after the bone metastasis diagnosis for any of the three cardiovascular events (composite event) and for each cardiovascular event separately.

### 2.4 Mortality

Information on date of death was obtained from the Danish Civil Registration System. This registry is updated daily and contains complete information on death and emigration since 1968.^16^

### 2.5 Other variables

Patient characteristics were extracted from the Civil Registration System and the Danish National Patient Registry. As a measure of comorbidity, we used the Charlson Comorbidity Index score.^24^ This score was calculated using information from the time of registration in the Danish National Patient Registry (as early as 1978) to the time of bone metastasis diagnosis. We used both primary and secondary diagnoses from in‐ and outpatient visits preceding the bone metastasis diagnosis. The index cancer diagnosis was not included in the calculation of the Charlson Comorbidity Index score.

To obtain information on antiplatelet and anticoagulation therapy, we linked our cohort to the Danish National Health Service Prescription Database.^25^ This registry has been operational since 2004 and contains information on all prescriptions registered using the Anatomical Therapeutic Chemical Classification System (ATC). All ICD codes and ATC codes used in this study are listed in Table S1. We used information on all drugs prescribed from 180 days before to 180 days after the date of bone metastasis diagnosis.

### 2.6 Statistical analysis

Patient characteristics at time of bone metastasis diagnosis were tabulated for the entire cohort and separately for each of the cohorts of patients with prostate cancer, breast cancer, lung cancer, and other cancers.

### 2.7 Risk of cardiovascular events and all‐cause mortality

For the risk analyses, we followed the cohort from the date of bone metastasis diagnosis until death, emigration, November 30, 2013, or a first‐time hospitalization or outpatient clinic visit for any cardiovascular event. Each of the three outcomes was also analyzed separately. We estimated incidence rates (IR) per 100 person‐years with 95% confidence intervals (CIs) and cumulative incidence with death as a competing risk for the cardiovascular events.^26^ The IRs were estimated for the time periods 0–30 days, 0–1 year, and 0–5 years after bone metastasis diagnosis, and cumulative incidences were estimated at 30 days, 1 year, and 5 years after diagnosis, and plotted for up to 5 years of follow‐up. We used the Kaplan–Meier method to calculate all‐cause mortality. All estimations were performed for the entire cohort and stratified by cancer type. As a sensitivity analysis to estimate the impact of pre‐existing heart disease, we estimated IRs and cumulative incidences after restriction to individuals that had not been registered with a cardiovascular event before the date of diagnosis of bone metastases.

### 2.8 Mortality following cardiovascular events

In this cohort analysis, the predictor was cardiovascular event and the outcome was all‐cause mortality. Cardiovascular events were included as a time‐varying variable where patients were followed from the time of bone metastasis diagnosis, at which point in time the patient started contributing risk time to the risk period without a cardiovascular event and then, if a cardiovascular event occurred, the patient would begin to contribute risk time to the risk period after the cardiovascular event. Patients were followed until death from any cause, emigration, or November 30, 2013, whichever came first. We used the Cox regression to estimate hazard ratios (HR) for death with 95% CIs comparing the two risk periods. HRs were adjusted for age at bone metastasis diagnosis, sex, and Charlson Comorbidity Index score. Analyses were performed using SAS V. 9.4 (SAS Institute Inc.) and R version 3.6.1 (R Foundation for Statistical Computing). The content of this paper followed the Strengthening of the Reporting of Observational Studies in Epidemiology (STROBE) guidelines^27^ and the Reporting of studies Conducted using Observational Routinely collected Data (RECORD) guidelines.^28^ The study was approved by the Danish Data Protection Agency (Aarhus University record no. 2016‐051‐000001, id 457).

## 3 RESULTS

### 3.1 Study population characteristics

The study population comprised 23,113 cancer patients with bone metastases (Table 1). Of these, 7040 (31%) had prostate cancer, 5315 (23%) had breast cancer, 3984 (17%) had lung cancer, and 6774 (29%) had other cancers. Among the patients with other cancers, 1833 had more than one cancer diagnosis including 668 patients with one of the cancers being prostate cancer, 586 patients with breast cancer as one of the cancers, and 460 patients with lung cancer as one of the cancers. In the overall cohort, there were slightly more males than females, and approximately half of the patients were <70 years old at the time of bone metastasis diagnosis. The follow‐up period ranged from 1 day to 19 years and 11 months. Antiplatelet and anticoagulation therapy were only registered for patients included from 2004 onwards (*n* = 13,308). In this period, the proportion of patients prescribed with anticoagulation therapy was high (73%). Aspirin was most commonly used (64.9%), while vitamin‐K antagonists (6.8%) and low‐molecular‐weight heparins (5.8%) were administered in a minority of patients.

**TABLE 1:** Characteristics at time of bone metastasis diagnosis among 23,113 cancer patients

	All patients *n* (%)	Prostate cancer *n* (%)	Breast cancer *n* (%)	Lung cancer *n* (%)	Other cancers *n* (%)
Age at bone metastasis diagnosis					
<60 years	5318 (23.0)	463 (6.6)	1926 (36.2)	1088 (27.3)	1841 (27.2)
60–69 years	6916 (29.9)	1770 (25.1)	1603 (30.2)	1500 (37.7)	2043 (30.2)
≥70 years	10,879 (47.1)	4807 (68.3)	1786 (33.6)	1396 (35.0)	2890 (42.7)
Age at cancer diagnosis					
<60 years	7439 (32.2)	744 (10.6)	2896 (54.5)	1166 (29.3)	2633 (38.9)
60–69 years	7298 (31.6)	2327 (33.1)	1339 (25.2)	1510 (37.9)	2122 (31.3)
≥70 years	8376 (36.2)	3969 (56.4)	1080 (20.3)	1308 (32.8)	2019 (29.8)
Male sex	13,219 (57.2)	7040 (100.0)	22 (0.4)	2263 (56.8)	3894 (57.5)
Year of bone metastasis diagnosis					
1994–2000	4884 (21.1)	1652 (23.5)	1219 (22.9)	720 (18.1)	1293 (19.1)
2001–2005	6917 (29.9)	2248 (31.9)	1699 (32.0)	1077 (27.0)	1893 (27.9)
2006–2010	6640 (28.7)	1954 (27.8)	1486 (28.0)	1157 (29.0)	2043 (30.2)
2011–2013	4672 (20.2)	1186 (16.8)	911 (17.1)	1030 (25.9)	1545 (22.8)
Year of primary cancer diagnosis					
1978–2000	9335 (40.4)	2757 (39.2)	2994 (56.3)	851 (21.4)	2733 (40.3)
2001–2005	6480 (28.0)	2326 (33.0)	1248 (23.5)	1072 (26.9)	1834 (27.1)
2006–2010	5263 (22.8)	1597 (22.7)	879 (16.5)	1208 (30.3)	1579 (23.3)
2011–2013	2035 (8.8)	360 (5.1)	194 (3.7)	853 (21.4)	628 (9.3)
CCI score (excluding cancer)					
0	14,782 (64.0)	4241 (60.2)	3998 (75.2)	2274 (57.1)	4269 (63.0)
1–2	6936 (30.0)	2287 (32.5)	1140 (21.4)	1438 (36.1)	2071 (30.6)
≥3	1395 (6.0)	512 (7.3)	177 (3.3)	272 (6.8)	434 (6.4)

Abbreviation: CCI, Charlson Comorbidity Index.

### 3.2 Risk of all‐cause mortality and cardiovascular events

All‐cause mortality was 26.6% at 30 days, 72.4% at 1 year, and 95.0% at 5 years after bone metastasis diagnosis. Table 2 presents the IRs and cumulative incidences for any cardiovascular event and for each separate cardiovascular event. In the overall cohort, the IR for any cardiovascular event was highest within 0–30 days after the diagnosis of bone metastasis and was reduced to approximately one third when looking at 0–5 years of follow‐up. Lung cancer patients had the highest IRs of cardiovascular events. For all cancer types, the IR after 5 years of follow‐up was only slightly lower than after 1 year of follow‐up. The diagnosis of myocardial infarction and ischemic stroke was predominately made during hospital admission, only 3% of the diagnoses of myocardial infarction and 11% of the diagnoses of ischemic stroke was made following out‐patient clinic visits.

**TABLE 2:** Incidence rates and cumulative incidences of cardiovascular events among 23,113 cancer patients with bone metastases

	Follow‐up period	Outcome	Incidence rates per 100 person‐years (95% CI)	Cumulative incidence, % (95% CI)
All patients *n* = 23,113	0–30 days	Any cardiovascular event	19.1 (17.0–21.3)	1.32 (1.18–1.48)
		Myocardial infarction	3.8 (2.9–4.9)	0.26 (0.20–0.34)
		Ischemic stroke	5.1 (4.1–6.3)	0.36 (0.29–0.44)
		Venous thromboembolism	10.6 (9.1–12.3)	0.74 (0.63–0.85)
	0–1 year	Any cardiovascular event	8.2 (7.7–8.8)	3.65 (3.41–3.90)
		Myocardial infarction	1.5 (1.3–1.7)	0.67 (0.57–0.78)
		Ischemic stroke	2.3 (2.0–2.6)	1.03 (0.91–1.17)
		Venous thromboembolism	4.6 (4.2–5.0)	2.06 (1.88–2.25)
	0–5 years	Any cardiovascular event	6.1 (5.8–6.5)	5.21 (4.92–5.51)
		Myocardial infarction	1.1 (0.9–1.2)	0.95 (0.83–1.09)
		Ischemic stroke	1.8 (1.6–2.0)	1.62 (1.46–1.80)
		Venous thromboembolism	3.3 (3.0–3.5)	2.81 (2.60–3.04)
Prostate cancer *n* = 7040	0–30 days	Any cardiovascular event	20.5 (17.0–24.8)	1.51 (1.24–1.81)
		Myocardial infarction	6.3 (4.5–8.9)	0.47 (0.33–0.65)
		Ischemic stroke	5.8 (4.0–8.3)	0.43 (0.30–0.60)
		Venous thromboembolism	8.9 (6.6–11.8)	0.65 (0.49–0.87)
	0–1 year	Any cardiovascular event	8.2 (7.3–9.2)	4.25 (3.80–4.75)
		Myocardial infarction	2.3 (1.8–2.8)	1.20 (0.96–1.48)
		Ischemic stroke	2.8 (2.3–3.4)	1.50 (1.23–1.81)
		Venous thromboembolism	3.2 (2.7–3.8)	1.69 (1.40–2.01)
	0–5 years	Any cardiovascular event	6.6 (6.0–7.2)	6.51 (5.93–7.12)
		Myocardial infarction	1.8 (1.5–2.1)	1.83 (1.53–2.17)
		Ischemic stroke	2.6 (2.2–3.0)	2.64 (2.28–3.05)
		Venous thromboembolism	2.3 (2.0–2.7)	2.31 (1.97–2.70)
Breast cancer *n* = 5315	0–30 days	Any cardiovascular event	15.4 (12.0–19.8)	1.13 (0.87–1.44)
		Myocardial infarction	1.5 (0.7–3.4)	0.11 (0.05–0.24)
		Ischemic stroke	3.3 (1.9–5.7)	0.24 (0.14–0.41)
		Venous thromboembolism	10.5 (7.7–14.3)	0.77 (0.56–1.04)
	0–1 year	Any cardiovascular event	5.3 (4.6–6.2)	3.14 (2.69–3.64)
		Myocardial infarction	0.5 (0.3–0.8)	0.31 (0.18–0.49)
		Ischemic stroke	1.3 (0.9–1.7)	0.77 (0.56–1.03)
		Venous thromboembolism	3.6 (3.0–4.3)	2.12 (1.76–2.54)
	0–5 years	Any cardiovascular event	4.0 (3.6–4.6)	5.43 (4.82–6.10)
		Myocardial infarction	0.4 (0.2–0.5)	0.50 (0.33–0.74)
		Ischemic stroke	1.0 (0.8–1.3)	1.36 (1.06–1.72)
		Venous thromboembolism	2.7 (2.3–3.1)	3.65 (3.15–4.20)
Lung cancer *n* = 3984	0–30 days	Any cardiovascular event	27.6 (21.7–35.1)	1.69 (1.32–2.12)
		Myocardial infarction	3.7 (1.9–7.1)	0.23 (0.11–0.42)
		Ischemic stroke	6.5 (4.0–10.7)	0.40 (0.24–0.64)
		Venous thromboembolism	18.5 (13.8–24.7)	1.13 (0.84–1.50)
	0–1 year	Any cardiovascular event	14.8 (12.6–17.3)	3.84 (3.27–4.47)
		Myocardial infarction	1.7 (1.1–2.7)	0.46 (0.28–0.72)
		Ischemic stroke	3.9 (2.8–5.3)	1.03 (0.75–1.39)
		Venous thromboembolism	9.6 (7.9–11.7)	2.50 (2.05–3.03)
	0–5 years	Any cardiovascular event	13.3 (11.4–15.3)	4.35 (3.73–5.03)
		Myocardial infarction	1.4 (0.9–2.2)	0.46 (0.28–0.72)
		Ischemic stroke	3.3 (2.5–4.5)	1.13 (0.83–1.50)
		Venous thromboembolism	8.9 (7.4–10.6)	2.95 (2.45–3.53)
Other cancers *n* = 6774	0–30 days	Any cardiovascular event	16.0 (12.7–20.1)	1.06 (0.84–1.33)
		Myocardial infarction	2.9 (1.7–4.9)	0.19 (0.11–0.32)
		Ischemic stroke	5.1 (3.4–7.6)	0.34 (0.22–0.50)
		Venous thromboembolism	8.4 (6.1–11.6)	0.56 (0.41–0.76)
	0–1 year	Any cardiovascular event	9.1 (8.0–10.4)	3.32 (2.90–3.77)
		Myocardial infarction	1.4 (1.0–2.0)	0.53 (0.37–0.73)
		Ischemic stroke	2.0 (1.5–2.7)	0.75 (0.57–0.98)
		Venous thromboembolism	5.8 (5.0–6.9)	2.14 (1.81–2.51)
	0–5 years	Any cardiovascular event	6.5 (5.8–7.4)	4.17 (3.69–4.69)
		Myocardial infarction	1.0 (0.7–1.4)	0.67 (0.49–0.90)
		Ischemic stroke	1.5 (1.2–1.9)	1.01 (0.79–1.29)
		Venous thromboembolism	4.0 (3.5–4.7)	2.61 (2.24–3.03)

Note

Any cardiovascular event: myocardial infarction, ischemic stroke, or venous thromboembolism.

Abbreviation: CI, confidence interval.

**FIGURE 2:**
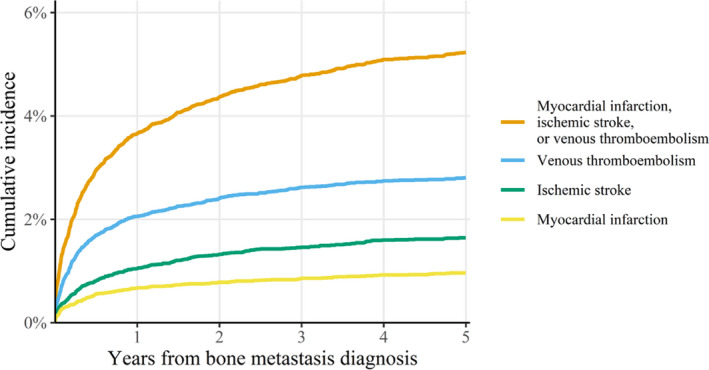
Cumulative incidence with death as a competing risk for cardiovascular events among 23,113 cancer patients with bone metastases

Looking at each of the cardiovascular events, the IRs were highest for VTE. Yet, for prostate cancer patients, the IR for ischemic stroke was slightly higher after 5 years of follow‐up. The IR for ischemic stroke was generally higher than for myocardial infarction. Figure 2 shows the cumulative incidence of cardiovascular events combined and for the three separate events. The 5‐year cumulative incidence was highest for VTE for all cancer types, except for prostate cancer, where the cumulative incidence was highest for ischemic stroke. Restriction to patients that had not had a cardiovascular event before the bone metastasis diagnosis (*n* = 20,030) reduced the estimates for IR and cumulative incidence, but only slightly (Table S2).

### 3.3 Mortality following cardiovascular events

HRs comparing mortality in the risk period until and after development of a cardiovascular event are presented in Table 3. Development of a cardiovascular event was a strong predictor of death at any time during follow‐up for all cancer types. For the overall cohort and following the cardiovascular event, the largest impact on mortality was during the first 30 days of follow‐up, but the decrease from 0–30 days of follow‐up to 0–5 years of follow‐up was minor (2.09–1.79). Myocardial infarction had the largest impact on mortality within the first 30 days of follow‐up. After 30 days, ischemic stroke was the event with the largest impact on mortality rates. Cardiovascular events had the largest impact on mortality among patients with prostate cancer. However, for 0–5 years of follow‐up, the estimates were similar to those of breast cancer patients.

**TABLE 3:** Hazard ratios for death among 23,113 cancer patients with bone metastases, comparing mortality in the risk period after development of cardiovascular event versus the risk period until developing cardiovascular event

Population	Follow‐up period	Exposure	Death during risk period until cardiovascular event, *N*	Death during risk period after cardiovascular event, *N*	Adjusted hazard ratio (95% CI)
All patients *n* = 23,113	0–30 days	Any cardiovascular event	6075	96	2.09 (1.71–2.56)
		Myocardial infarction	6147	24	2.71 (1.81–4.05)
		Ischemic stroke	6142	29	2.03 (1.41–2.92)
		Venous thromboembolism	6127	44	1.86 (1.38–2.50)
	0–1 year	Any cardiovascular event	15,967	579	1.84 (1.70–2.01)
		Myocardial infarction	16,432	114	1.88 (1.56–2.26)
		Ischemic stroke	16,372	174	2.10 (1.81–2.44)
		Venous thromboembolism	16,233	313	1.71 (1.53–1.91)
	0–5 years	Any cardiovascular event	19,894	980	1.79 (1.67–1.91)
		Myocardial infarction	20,689	185	1.82 (1.58–2.11)
		Ischemic stroke	20,571	303	1.81 (1.62–2.03)
		Venous thromboembolism	20,346	528	1.74 (1.59–1.89)
Prostate cancer *n* = 7040	0–30 days	Any cardiovascular event	1350	39	3.66 (2.66–5.04)
		Myocardial infarction	1375	14	3.93 (2.32–6.66)
		Ischemic stroke	1377	12	3.31 (1.88–5.86)
		Venous thromboembolism	1375	14	3.71 (2.19–6.28)
	0–1 year	Any cardiovascular event	4455	205	2.07 (1.80–2.39)
		Myocardial infarction	4596	64	2.26 (1.76–2.89)
		Ischemic stroke	4583	77	2.28 (1.82–2.86)
		Venous thromboembolism	4588	72	1.76 (1.39–2.22)
	0–5 years	Any cardiovascular event	5953	388	1.93 (1.74–2.14)
		Myocardial infarction	6230	111	2.10 (1.74–2.54)
		Ischemic stroke	6184	157	2.12 (1.80–2.48)
		Venous thromboembolism	6204	137	1.63 (1.37–1.93)
Breast cancer *n* = 5315	0–30 days	Any cardiovascular event	1018	12	1.75 (0.99–3.10)
		Myocardial infarction	1029	1	1.58 (0.22–11.21)
		Ischemic stroke	1026	4	2.18 (0.81–5.86)
		Venous thromboembolism	1023	7	1.60 (0.76–3.36)
	0–1 year	Any cardiovascular event	2819	92	2.04 (1.66–2.52)
		Myocardial infarction	2901	10	2.11 (1.13–3.93)
		Ischemic stroke	2885	26	2.77 (1.88–4.10)
		Venous thromboembolism	2853	58	1.80 (1.39–2.34)
	0–5 years	Any cardiovascular event	4300	218	1.94 (1.69–2.23)
		Myocardial infarction	4499	19	1.51 (0.96–2.38)
		Ischemic stroke	4459	59	2.03 (1.57–2.63)
		Venous thromboembolism	4374	144	1.89 (1.60–2.24)
Lung cancer *n* = 3984	0–30 days	Any cardiovascular event	1582	20	1.33 (0.86–2.07)
		Myocardial infarction	1600	2	1.38 (0.34–5.55)
		Ischemic stroke	1597	5	1.29 (0.54–3.11)
		Venous thromboembolism	1589	13	1.27 (0.74–2.20)
	0–1 year	Any cardiovascular event	3460	128	1.66 (1.39–1.99)
		Myocardial infarction	3573	15	1.45 (0.87–2.41)
		Ischemic stroke	3553	35	2.19 (1.57–3.06)
		Venous thromboembolism	3505	83	1.49 (1.20–1.85)
	0–5 years	Any cardiovascular event	3680	155	1.60 (1.36–1.88)
		Myocardial infarction	3818	17	1.44 (0.89–2.32)
		Ischemic stroke	3795	40	1.74 (1.27–2.38)
		Venous thromboembolism	3730	105	1.55 (1.28–1.89)
Other cancers *n* = 6774	0–30 days	Any cardiovascular event	2125	25	1.68 (1.13–2.50)
		Myocardial infarction	2143	7	2.83 (1.35–5.96)
		Ischemic stroke	2142	8	1.52 (0.76–3.05)
		Venous thromboembolism	2140	10	1.35 (0.72–2.51)
	0–1 year	Any cardiovascular event	5233	154	1.39 (1.18–1.63)
		Myocardial infarction	5362	25	1.40 (0.94–2.08)
		Ischemic stroke	5351	36	1.42 (1.02–1.98)
		Venous thromboembolism	5287	100	1.40 (1.14–1.70)
	0–5 years	Any cardiovascular event	5961	219	1.39 (1.21–1.59)
		Myocardial infarction	6142	38	1.53 (1.11–2.10)
		Ischemic stroke	6133	47	1.13 (0.85–1.51)
		Venous thromboembolism	6038	142	1.48 (1.25–1.75)

Note

Cardiovascular events were included in the analyses as a time‐dependent exposure. Hazard ratios were adjusted for age at time of bone metastasis diagnosis, sex, and Charlson Comorbidity Index score. Any cardiovascular event: myocardial infarction, ischemic stroke, or venous thromboembolism

Abbreviation: CI, confidence interval.

## 4 DISCUSSION

This population‐based cohort study with inclusion of more than 20,000 patients is the first to investigate the risk of cardiovascular events among cancer patients with bone metastases. Cardiovascular events occurred in 4% of patients within 1 year of follow‐up despite a strong competing risk of death, with VTE being the most common event. Although the all‐cause mortality was considerable, development of cardiovascular events was associated with an approximately two‐fold increase in mortality compared to patients without cardiovascular disease during up to 5 years of follow‐up.

The association between cancer and arterial thromboembolism has recently been evaluated in a meta‐analysis.^29^ The risk of myocardial infarction and stroke was elevated among cancer patients, with the highest risk occurring just after bone metastasis diagnosis, and with risk being associated with cancer type. Only one of the included studies analyzed information on cancer stage in relation to the risk of arterial events.^30^ In that study, including 279,719 American cancer patients, the relative risks of myocardial infarction and ischemic stroke increased from 1.0 in stage 1 cancer to 1.8 in stage 4 cancer 6–9 months after cancer diagnosis compared to matched cancer‐free individuals. One year after cancer diagnosis, the cumulative incidence was 4.7% for stage 1 patients and 9.4% for stage 4 (metastatic cancer) patients.^30^ Myocardial infarction and ischemic stroke combined led to a four‐fold increase in the relative hazard for mortality. The excess mortality due to arterial events was not evaluated by cancer stage but it emphasizes that even in a population with high mortality rates, relative mortality rates can increase substantially with an arterial event. Our cohort had very high mortality rates, but still ischemic stroke and myocardial infarction had substantial impact on mortality. These results indicate a need for increased focus on optimal prevention and treatment for stroke and myocardial infarction among cancer patients with bone metastases.^31^

It is well‐known that VTE is a major cause of death in cancer patients.^32^ Cancer‐related risk factors for VTE include cancer type, histological grade, primary site, and stage.^33^ While cancer stage is an important predictor for the development of VTE in some studies,^34^, ^35^ it is unrelated in others.^36^ No study has examined the risk of VTE among stage 4 cancer patients, i.e., patients with distant metastases. VTE was the most common cardiovascular event in our study. Although direct comparison is not possible due to methodological differences, primarily the inclusion of hospitalized patients only, it is interesting that the incidence of VTE in our study is almost 8 times higher than in an otherwise comparable Danish cohort of general cancer patients.^37^ This indicates that bone metastasis could be an important risk factor for the development of VTE. There may be several biological explanations for this, including patient related factors like increased immobilization and permanent catheters, and factors directly related to the metastasized cancer like increased expression of or release of tissue factor from cancer cells and compression of veins from tumor masses. The prognostic impact on mortality was less pronounced for VTE compared with myocardial infarction and ischemic stroke.

The presented results should be interpreted in light of certain limitations. Generally, the quality of the Danish registries is high. Yet, compared with medical charts the completeness of bone metastasis registrations in the Danish National Patient Registry is approximately 40% with a positive predictive value of 90%.^20^ With no formal procedures for detecting bone metastases, they likely remain undiagnosed in patients with a poor prognosis where the clinical implications of the finding is limited, or opposite, they may remain undiagnosed in patients with asymptomatic bone metastases that may have a better prognosis. How this affects the composition of the study population compared to the target population is not possible to answer. Further, we had information on community‐filled prescriptions of anticoagulant treatment, but not on treatment administered during hospital admission and thus the number of patients receiving anticoagulant treatment is presumably underreported in our study, particularly for low‐molecular‐weight heparin. This issue prevented us from conducting detailed analyses according to anticoagulant treatment. Lastly, in the analysis of mortality following cardiovascular disease, some cardiovascular deaths may occur without the patient reaching the hospital. In these cases, the cardiovascular disease will not be captured which will result in an underestimation of the mortality following cardiovascular disease. We believe that this is a rather small number of cases, and that the resulting bias therefore is neglectable.

In our analyses, we accounted for the competing risk of death. Despite the fact that the mortality, as expected, was high, cardiovascular events were frequent. We only considered the first inpatient hospitalization or outpatient clinic visit after bone metastases diagnosis for the cardiovascular event, and did not take into account the individual cardiovascular events as a potential competing risk, as one cardiovascular event could be on the causal pathway to a subsequent cardiovascular event. For example, patients with ischemic stroke or myocardial infarction are at increased risk of VTE. Besides, arterial events have also been associated with an increased risk of VTE.^38^ Another potential source of bias in this analysis is that the time of cardiovascular disease may be related to cancer progression and, hence, to death. This may lead to an overestimation of the effect of cardiovascular disease on death.

## 5 CONCLUSION

Cardiovascular events occurred in approximately 4% of cancer patients with bone metastases within 1 year from diagnosis, with comparable estimates across cancer types. Rates of cardiovascular disease were highest in the first 30 days following diagnosis of bone metastases. The risk was consistently higher for VTE compared to ischemic stroke and myocardial infarction. Despite being a population with a very high baseline mortality, cardiovascular events doubled mortality among cancer patients with bone metastases.

## CONFLICT OF INTEREST

AS and RH are employees of and own shares in Amgen. TR, HTS, AP, PA, KA, and JS have no personal conflict of interest.

## Supporting information

Table S1‐S2
