# Supplementary material for: Cardiovascular events in cancer patients with bone metastases—A Danish population‐based cohort study of 23,113 patients
Source: Cancer Med. 2021 Jun 2;10(14):4885–95. doi: 10.1002/cam4.4027 (PMC8290242; doi:10.1002/cam4.4027)
Supplement: Supplementary file 1 — Table S1‐S2 [file CAM4-10-4885-s001.docx]

| **Supplementary Table 1.** Diagnosis and procedure codes | |
| --- | --- |
|  | **Diagnosis and procedure codes**  According to ICD-10 unless otherwise specified |
| Bone metastasis | C79.5 |
| **Cardiovascular events** |  |
| Myocardial infarction | I21, I22, I23 |
| Ischemic stroke | I63, I64, G459 |
| Venous thromboembolism | I26, I80.1—I80.3, I81, I820, I822, I823, I808B, I828, I829, K550H |
| **Cancer types** |  |
| Hematological malignancies | C81, C82, C83, C84, C85, C86, C87, C88, C89, C90, C91, C92, C93, C94, C95, C96 |
| Prostate | C61 |
| Breast | C50 |
| Lung | C33, C34, C38, C39 |
| Intestine incl. colon and rectum | C17, C18, C19, C26, C20, C21 |
| Urinary tract cancers incl. kidneys | C64, C65, C66, C67, C68 |
| Metastases and non-specified cancer | C76, C77, C78, C79 |
| Other | C0, C10, C11, C12, C13, C14, C30, C31, C32, C73, C74, C75, C69, C70, C71, C72, C60, C62, C63, C53, C54, C55, C51, C52, C56, C57, C45, C46, C47, C48, C49, C25, C22, C23, C24, C15, C16 |
| **Charlson Comorbidity Score** |  |
| Myocardial infarction | DI21, DI22, DI23 + ICD-8: 410 |
| Congestive heart failure | DI50, DI110, DI130, DI132 + ICD-8: 42709, 42710, 42711, 42719, 42899, 78249 |
| Peripheral vascular disease | DI70, DI71, DI72, DI73, DI74, DI77 + ICD-8: 440, 441, 442, 443, 444, 445 |
| Cerebrovascular disease | DI6, DG45, DG46 + ICD-8: 430, 431, 432, 433, 434, 435, 436, 437, 438 |
| Dementia | DF00, DF01, DF02, DF03, DF051, DG30 + ICD-8: 29009, 2901, 29309 |
| Chronic pulmonary disease | DJ40, DJ41, DJ42, DJ43, DJ44, DJ45, DJ46, DJ47, DJ60, DJ61, DJ62, DJ63, DJ64, DJ65, DJ66, DJ67, DJ684, DJ701, DJ703, DJ841, DJ920, DJ961, DJ982, DJ983 + ICD-8: 490, 491, 492, 493, 515, 516, 517, 518 |
| Connective tissue disease | DM05, DM06, DM08, DM09, DM30, DM31, DM32, DM33, DM34, DM35, DM36, DD86 + ICD-8: 712, 716, 734, 446, 13599 |
| Ulcer disease | DK221, DK25, DK26, DK27, DK28 + ICD-8: 53091, 53098, 531, 532, 533, 534 |
| Mild liver disease | DB18, DK700, DK701, DK702, DK703, DK709, DK71, DK73, DK74, DK760 + ICD-8: 571, 57301, 57304 |
| Diabetes without end-organ damage | DE100, DE101, DE109, DE110, DE111, DE119 + ICD-8: 24900, 24906, 24907, 24909, 25000, 25006, 25007, 25009 |
| Hemiplegia | DG81, DG82 + ICD-8: 344 |
| Moderate to severe renal disease | DI12, DI13, DN00, DN01, DN02, DN03, DN04, DN05, DN07, DN11, DN14, DN17, DN18, DN19, DQ61 + ICD-8: 403, 404, 580, 581, 582, 583, 584, 59009, 59319, 7531, 792 |
| Diabetes with end-organ damage | DE102, DE103, DE104, DE105, DE106, DE107, DE108, DE112, DE113, DE114, DE115, DE116, DE117, DE118 + ICD-8: 24901, 24902, 24903, 24904, 24905, 24908, 25001, 25002, 25003, 25004, 25005, 25008 |
| Non-metastatic solid tumour | DC0, DC1, DC2, DC3, DC4, DC5, DC6, DC70, DC71, DC72, DC73, DC74, DC75 + ICD-8: 14, 15, 16, 17, 18, 190, 191, 192, 193, 194 |
| Leukaemia | DC91, DC92, DC93, DC94, DC95 + ICD-8: 204, 205, 206, 207 |
| Lymphoma | DC81, DC82, DC83, DC84, DC85, DC88, DC90, DC96 + ICD-8: 200, 201, 202, 203, 27559 |
| Moderate to severe liver disease | DB150, DB160, DB162, DB190, DK704, DK72, DK766, DI85 + ICD-8: 07000, 07002, 07004, 07006, 07008, 57300, 4560 |
| Metastatic solid tumour | DC77, DC78, DC79, DC80 + ICD-8: 195, 196, 197, 198, 199, DC76 |
| AIDS | DB21, DB22, DB23, DB24 + ICD-8: 07983 |
| **Oral anticoagulation therapy** | ATC codes:  Vitamin-K antagonist: B01AA03, B01AA04  Non–vitamin K antagonist oral anticoagulants: MB01AF01, MB01AF02, MB01AF03, MB01AE07  Low-molecular-weight heparins: B01AB04, B01AB05, B01AB10  Aspirin: B01AC06, N02BA01, N02BA51, M01A (excl· M01AX05)  ADP blockers: B01AC04, B01AC22, B01AC24  Statins: C10AA01, C10AA02, C10AA05 |
| ICD-10: International Classification of Diseases 10^th^ Revision, ATC: Anatomical Therapeutic Chemical Classification System | |

| **Supplementary Table 2.** Incidence rates and cumulative incidences of cardiovascular events among 20,030 cancer patients with bone metastases who had not experienced a cardiovascular event before their bone metastasis diagnosis. | | | | |
| --- | --- | --- | --- | --- |
|  | **Follow-up period** | **Outcome** | **Incidence rates per 100 person-years (95% CI)** | **Cumulative incidence,**  **% (95% CI)** |
| **All patients**  **n=23,113** | **0–30 days** | **Any cardiovascular event** | **12.7 (10.9-14.7)** | **0.98 (0.77-1.03)** |
|  |  | Myocardial infarction | 2.6 (1.9-3.6) | 0.19 (0.13-0.25) |
|  |  | Ischemic stroke | 2.7 (2.0-3.7) | 0.19 (0.14-0.26) |
|  |  | Venous thromboembolism | 7.7 (6.4-9.3) | 0.54 (0.45-0.65) |
|  | **0–1 year** | **Any cardiovascular event** | **6.6 (6.1-7.2)** | **3.04 (2.81-3.29)** |
|  |  | Myocardial infarction | 1.1 (0.9-1.3) | 0.50 (0.41-0.61) |
|  |  | Ischemic stroke | 1.7 (1.5-2.0) | 0.80 (0.69-0.94) |
|  |  | Venous thromboembolism | 3.9 (3.5-4.3) | 1.80 (1.62-1.99) |
|  | **0–5 years** | **Any cardiovascular event** | **5.1 (4.7-5.4)** | **4.51 (4.22-4.81)** |
|  |  | Myocardial infarction | 0.8 (0.7-1.0) | 0.76 (0.64-0.90) |
|  |  | Ischemic stroke | 1.5 (1.3-1.7) | 1.35 (1.19-1.53) |
|  |  | Venous thromboembolism | 2.8 (2.6-3.1) | 2.53 (2.31-2.76) |
